# Frequent copy number variants in a cohort of Mexican-Mestizo individuals

**DOI:** 10.1186/s13039-022-00631-z

**Published:** 2023-01-12

**Authors:** Silvia Sánchez, Ulises Juárez, Julieta Domínguez, Bertha Molina, Rehotbevely Barrientos, Angélica Martínez-Hernández, Alessandra Carnevale, Patricia Grether-González, Dora Gilda Mayen, Camilo Villarroel, Esther Lieberman, Emiy Yokoyama, Victoria Del Castillo, Leda Torres, Sara Frias

**Affiliations:** 1 Laboratorio de Citogenética, Instituto Nacional de Pediatría, Insurgentes Sur 3700-C Insurgentes Cuicuilco, P01090 Ciudad de Mexico, México; 2 Posgrado en Ciencias Biológicas, Universidad Nacional Autónoma de México, Ciudad de México, México; 3 Laboratorio de Inmunogenómica y Enfermedades Metabólicas, Instituto Nacional de Medicina Genómica, Ciudad de Mexico, México; 4 Laboratorio de Enfermedades Mendelianas, Instituto Nacional de Medicina Genómica, Ciudad de Mexico, México; 5 Departamento de Genética y Genómica Humana, Instituto Nacional de Perinatología, Ciudad de Mexico, México; 6 Unidad de Genética Aplicada. Hospital Ángeles Lomas, Huixquilucan, Edo. de México México; 7 Genética Humana, Instituto Nacional de Pediatría, Ciudad de Mexico, México; 8 Departamento de Medicina Genómica y Toxicología Ambiental, Instituto de Investigaciones Biomédicas, Universidad Nacional Autónoma de México, Ciudad de México, México; 9 Present Address: Centro Médico ABC, Campus Santa Fe, Ciudad de Mexico, México

**Keywords:** CNV, Mexican population, Hispanic population, 2p11.2, 8p11.22, 14q32.33, 15q11.2, Affymetrix SNP6.0

## Abstract

**Background:**

The human genome presents variation at distinct levels, copy number variants (CNVs) are DNA segments of variable lengths that range from several base pairs to megabases and are present at a variable number of copies in human genomes. Common CNVs have no apparent influence on the phenotype; however, some rare CNVs have been associated with phenotypic traits, depending on their size and gene content. CNVs are detected by microarrays of different densities and are generally visualized, and their frequencies analysed using the HapMap as default reference population. Nevertheless, this default reference is inadequate when the samples analysed are from people from Mexico, since population with a Hispanic genetic background are minimally represented. In this work, we describe the variation in the frequencies of four common CNVs in Mexican-Mestizo individuals.

**Results:**

In a cohort of 147 unrelated Mexican-Mestizo individuals, we found that the common CNVs 2p11.2 (99.6%), 8p11.22 (54.5%), 14q32.33 (100%), and 15q11.2 (71.1%) appeared with unexpectedly high frequencies when contrasted with the HapMap reference (ChAS). Yet, while when comparing to an ethnically related reference population, these differences were significantly reduced or even disappeared.

**Conclusion:**

The findings in this work contribute to (1) a better description of the CNVs characteristics of the Mexican Mestizo population and enhance the knowledge of genome variation in different ethnic groups. (2) emphasize the importance of contrasting CNVs identified in studied individuals against a reference group that—as best as possible—share the same ethnicity while keeping this relevant information in mind when conducting CNV studies at the population or clinical level.

## Background

The human genome presents distinct variations: single nucleotide variants, insertion‒deletion of a few nucleotides, repetitive sequences of a variable number of nucleotides, and structural variants. The human genome is 3.1 giga base pairs (Gb) in size, distributed in 23 pairs of chromosomes so that every individual inherits one copy from each parent and has two copies or copy number (CN) CN = 2 of every *locus* in their cells (except for the X and Y *loci* in males). In 2004, two independent groups, Iafrate and Sebat [1, 2], described for the first time the genome-wide presence of large-scale copy number variations in the human genome; these variants involve gains or losses of several to hundreds of kilobases (kb) of genomic DNA among phenotypically normal individuals, and these copy number variants (CNVs) are an important source of human genomic variation [3]. Common CNVs have a minor allele frequency (MAF) > 5% and no apparent influence on the phenotype. However, some rare CNVs, with MAF < 1%, have been associated with phenotypic traits [4], and a few of them have shown clinical relevance depending on their size, gene content, or when overlap with genes that manifest haploinsufficiency (when CNV is CN = 1) or triplosensitivity (when CNV is CN > 2) [5, 6]. In some occasions, their interaction with additional genetic or environmental factors may influence whether CNVs have a detectable phenotypic effect [7, 8]. CNV represents a significant proportion of the total genetic variability in all human populations [9–11]. A CNV was initially defined as a DNA segment larger than one kb with a variable copy number compared to a reference genome [12]. Nevertheless, this term has extended to all quantitative variations in the genome, including tandem repeats, deletions, and duplications [13].

The accurate detection and interpretation of CNVs are essential for research and clinical diagnostics because CNVs may be a general population variant or associated with pathology [14]. Therefore, it has become increasingly important to detect the presence of common and harmless CNVs within different ethnic groups to avoid misinterpretation of pathological variants.

The Database of Genomic Variants (DGV) was initiated to provide a publicly accessible, comprehensive, and curated catalog of genomic variants [15]. The DGV comprises the CNVs and structural variations found in the genomes of control individuals from worldwide populations. However, only three studies in this database included individuals with Mexican ancestry, and they are the same in all three studies [16–18]. Furthermore, currently it only comprises individuals born in the US with parents and grandparents of Mexican origin, representing only 3% of the total Mexican population [19].

In general, this could represent an issue since the contribution of the ethnic group is diluted when the entire group is used as a reference.

Therefore, it is necessary to report information on this type, especially for underrepresented populations.

Several methodologies are commonly used for CNV genome-wide study; one of the most robust methods is microarray analysis, mainly when an array contains a high density of probes. Most CNV studies are performed with single nucleotide polymorphism (SNP) microarrays, which, in addition to providing information on CNV in the genome, also provide information at the allele level, highlighting the presence of regions/runs of homozygosity called long contiguous stretches of homozygosity (LCSH) and providing essential clues regarding parental relatedness (consanguinity), uniparental disomy, chromosomal recombination or rearrangements [20, 21].

This work describes the frequency of four common CNVs found using SNP microarrays in unrelated Mexican-Mestizo individuals, who were healthy or had aneuploidies, and in whom structural alterations in their genome was not suspected. The study highlights the frequency variation of these common CNVs depending on the ethnic origin of the population used as reference for comparison.

It is essential to recognize the ethnic differences in the distribution and frequency of CNVs, not only to recognize their contribution to structural chromosomal variation but also to ease recognition of possible associations between CNVs and phenotypic characteristics that may or may not be pathogenic.

## Results

We studied the presence of CNV in a population of 147 Mexican-Mestizo individuals, 50 phenotypically normal (MXM, 30 females and 20 males) and 97 patients with aneuploidies (MXM_A, 41 with trisomy 21, 28 with monosomy X, 11 with trisomy 13 and 17 with trisomy 18), which are part of a more extensive study that aims to find genomic differences among these groups. In this first study, we show four CNVs in common among all these groups, with a very high frequency in our population when contrasted with distant populations, as is the mix of the HapMap reference that includes African, Asian, and Caucasian individuals.

### Long contiguous stretches of homozygosity

To recognize the presence of inbreeding and consanguinity in our sample population, we analyzed the regions with homozygosity or long contiguous stretches of homozygosity (LCSH) with Affymetrix Genome-Wide Human SNP Array 6.0 (GW SNP 6.0). Eighteen of the 50 healthy controls (MXM) studied showed at least one autosomal region with LCSH. In the aneuploidy group (MXM_A), 73/97 presented at least one autosomal region with LCSH.

In addition, we analyzed 99 CEL files from 99 blood samples of Mexican women with breast cancer (MXM_WBC) present in public databases, where 75/99 presented at least one autosomal region with LCSH.

Neither of these LCSH regions overlaps with the CNV reported in this study, and more than 42% are common LCSHs, such as the 2q11.1q11.2, 11p11.2p12.1, 16p11.2p11.1, and 20q11.21q11.23 regions previously reported [22–24]. The inbreeding coefficient (F) calculated in the three groups supports the absence of inbreeding or consanguinity (Table 1).

**Table 1:** LSCH characteristics in the studied population

	% NAT Component	Total Autosomal LSCH (kb, Avg)	F (Avg)
MXM	67.8	351,930.646	0.01221543
MXM_WBC	NA	1,988,679.847	0.00069027
MXM_A	61.70	17,787.83589	0.0062265

*NAT* Native American,* LCSH* Long-contiguous stretches of homozygosity, *Avg* average, *NA* not available, *MXM* Mexican Mestizo, *MXM_WBC* Women Breast Cancer, *MXM _A* Mexican Mestizo with aneuploidy, *F* Inbreeding Coefficient, shows no kinship

### Ancestry

**Fig. 1:**
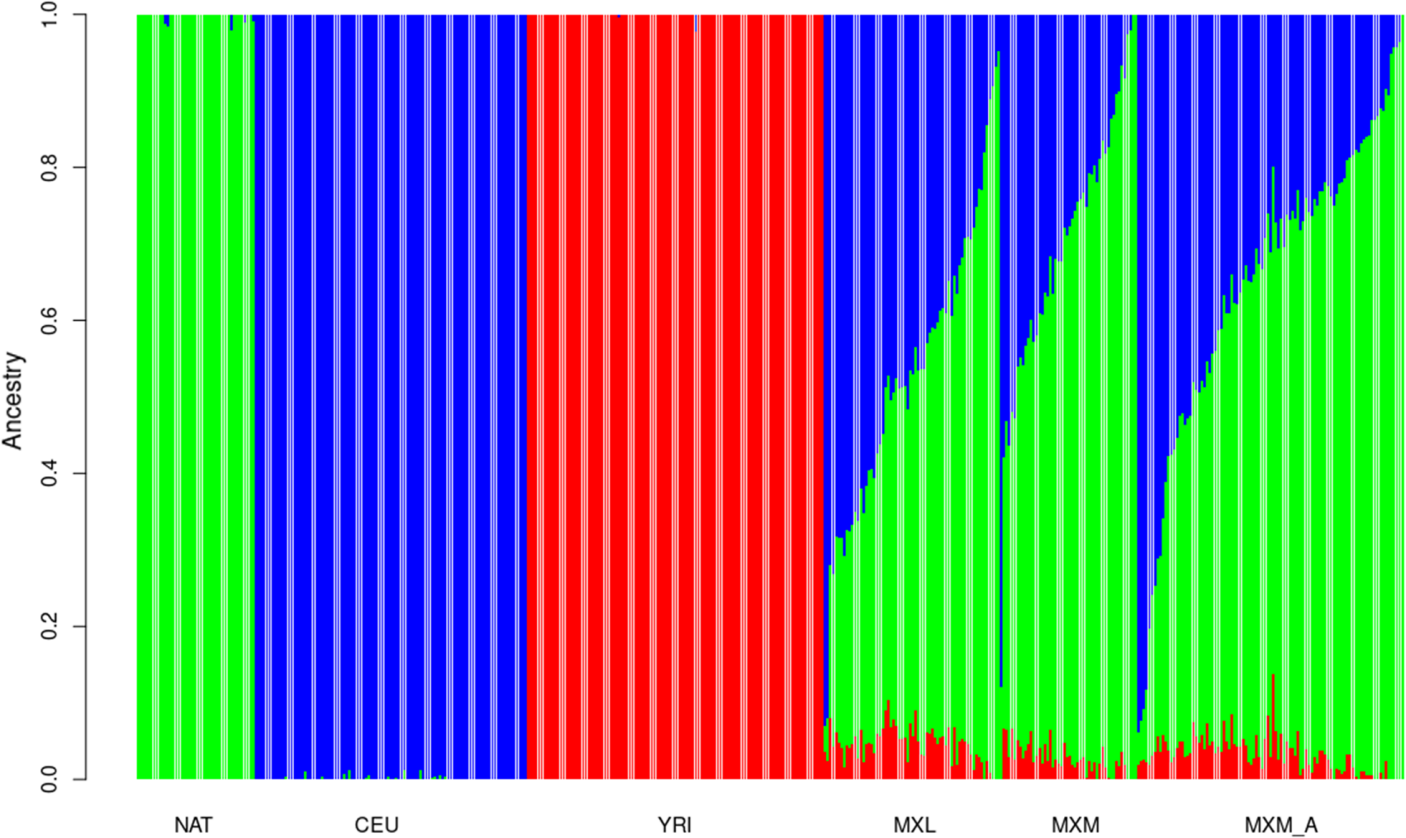
Ancestry pattern from each contributing population in the Mexican Mestizo Healthy (MXM) and Mexican Mestizo with aneuploidy (MXM_A) groups. Bar plot showing global ancestry of MXM groups deduced with ADMIXTURE. YRI: Yoruba in Ibadan, Nigeria; NAT: Native American; CEU: Caucasian

We deduced the ancestry in our MXM and MXM_A groups compared with the Mexican ancestry in Los Angeles CA, USA (MXL) included in the HapMap project (64 Mexican individuals born in the US). First, we noticed that our population (MXM and MXM_A) has almost the same genetic content of European descent from Utah, USA (CEU), Native American (NAT), and Yoruba from Nigeria (YRI), then those studied for the HapMap, with NAT being the most significant contribution, even greater than for MXL, 0.488 vs 0.678 and 0.618, then of Caucasians and finally Africans (Fig. 1).

### Copy number variants with high frequency in Mexican-Mestizo population

**Fig. 2:**
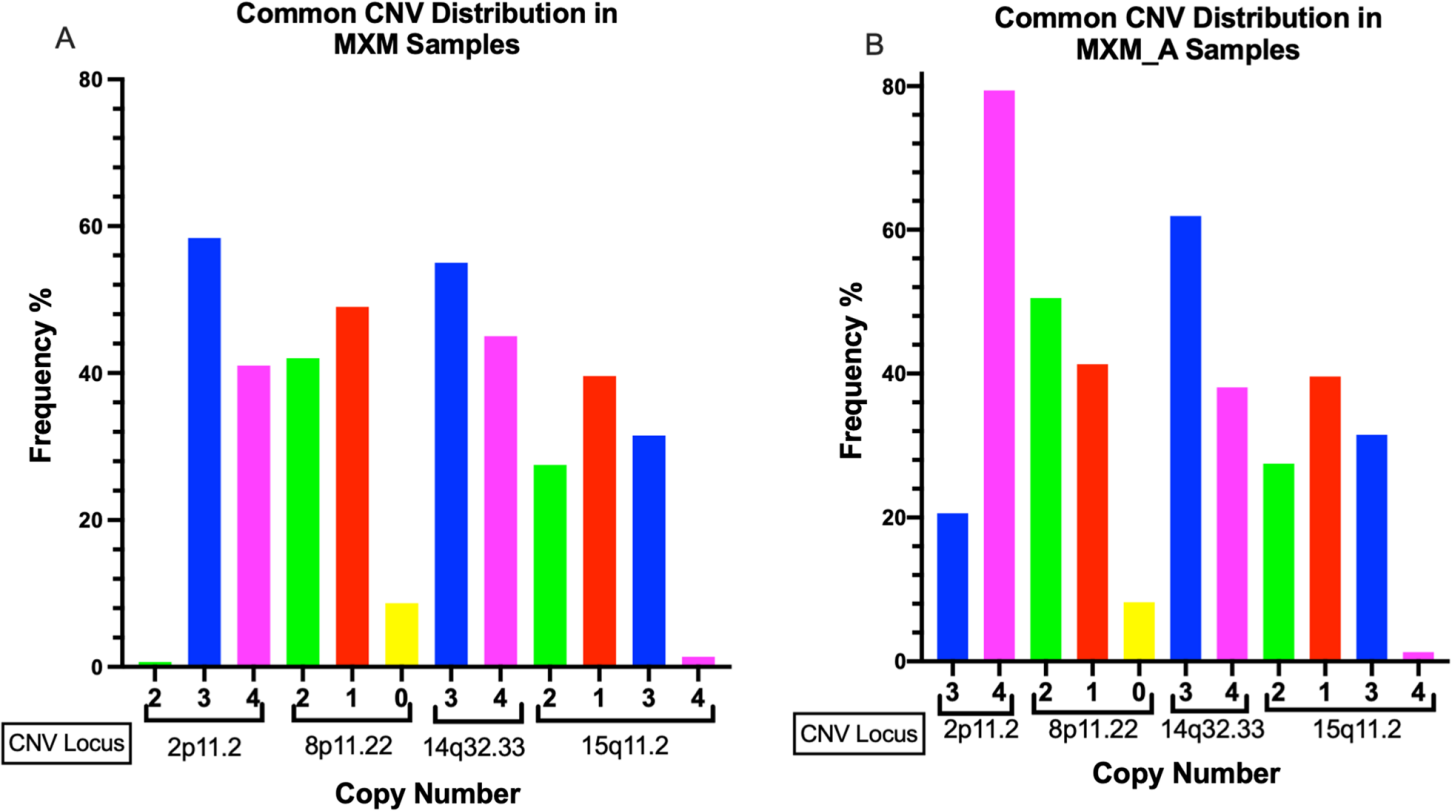
Distribution of copy number variants (CNVs) frequently found in the Mexican-Mestizo population. **a** Mexican-Mestizo Healthy (MXM). **b** Mexican-Mestizo with aneuploidy (MXM_A)

**Fig. 3:**
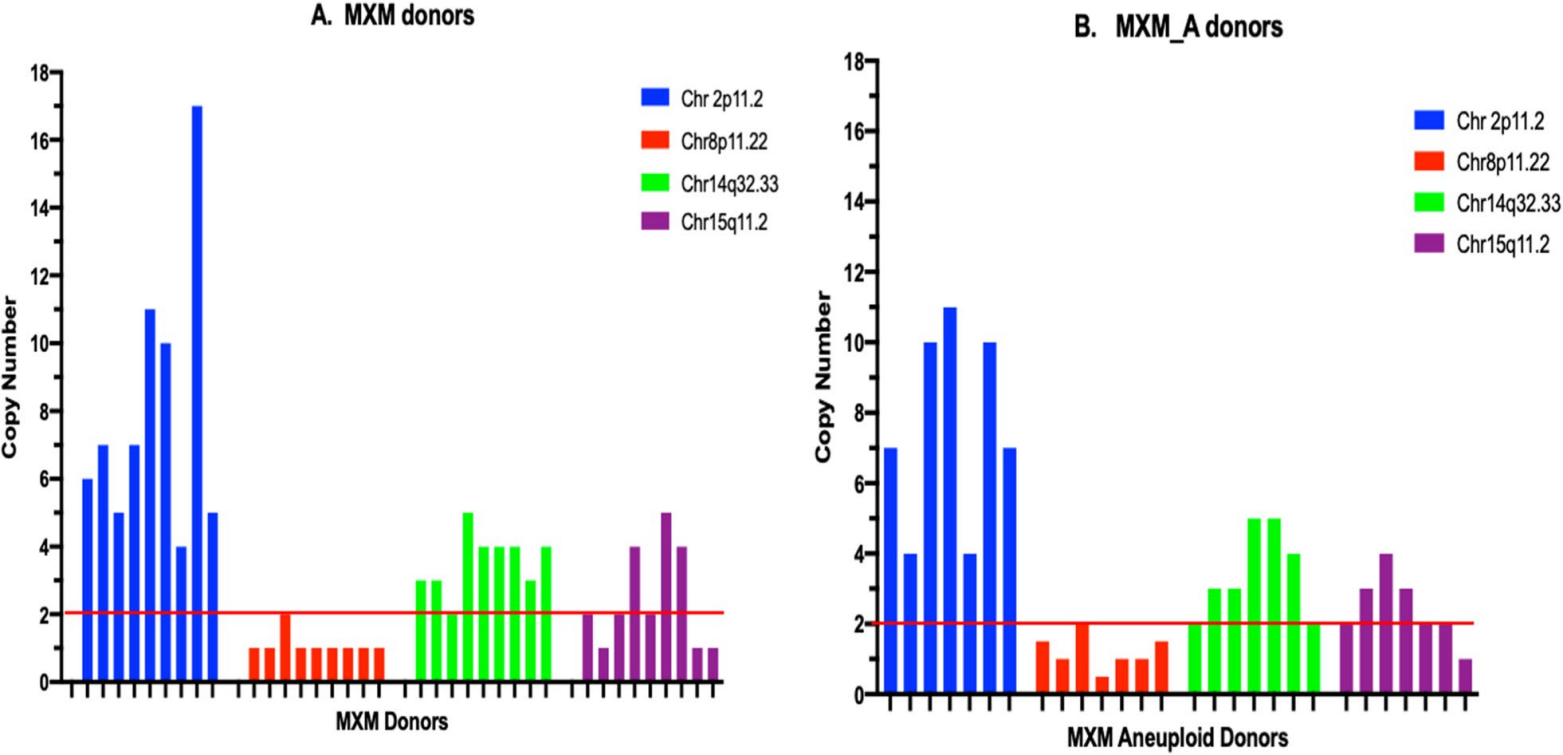
Validation of copy number variant (CNV) changes at four chromosome *loci* by qPCR. **a** Nine DNA samples from healthy Mexican-Mestizo (MXM) donors and **b** 7 DNA samples from Mexican-Mestizo donors with aneuploidy (MXM_A). The red line shows CN = 2

We found CNV in almost all individuals and in all the chromosomes; however, we observed four polymorphisms that occurred in chromosomes 2 (2.9 megabases (Mb)), 8 (0.15 Mb), 14 (1.02 Mb), and 15 (0.76 Mb) with a high frequency in the general population of Mexican-Mestizo (MXM), in Mexican-Mestizo with aneuploidy (MXM_A), and in the MXM_WBC group (Figs. 2 and 3) (Table 2).

**Table 2:** Copy number variants with high frequency in Mexican Mestizo individuals

*Locus*	CN	Healthy tissues				Aneuploid tissues		Total percentage across all studied population	Coordinates (hg19)	Genes located in the region
		MXM (n = 50)		MXM_WBC (n = 99)		MXM_A (n = 97)				
		n	Total (Percentage)	n	Total (Percentage)	n	Total (Percentage)			
2p11.2	3	25	49/50 (98%)	62	99/99 (100%)	20	97/97 (100%)	99.6%	chr2: 89,143,755–92,057,597. 2.9 Mb	*IGKV2D-28* (HGNC:5799), *IGKV3D-7* (HGNC:5829)
	4	24		37		77				
8p11.22	1	29	29/50 (58%)	44	57/99 (57.6%)	40	48/97 (49.5%)	54.5%	chr8: 39,230,170–39,386,952. 156,782 bp	*ADAM5* (HGNC: 212)*, ADAM3A* (HGNC:209)
	0	0		13		8				
14q32.33	3	20	50/50 (100%)	62	99/99 (100%)	55	97/97 (100%)	100%	chr14: 106,078,230–107,100,266. 1.02 Mb	*MIR4507* (HGNC:41,642)*, MIR4538* (HGNC:41,664)*, MIR4537* (HGNC:41,682)*, MIR4539* (HGNC:41,546), *KIAA0125* (HGNC:1995)*, ADAM6* (HGNC:213)*, LINC00226* (HGNC:20,168) *IGHD* (HGNC:5480) *IGHG3* (HGNC:5527) *IGHG1* (HGNC:5525)
	4	30		37		42				
15q11.2	1	21	45/50 (90%)	38	63/99 (63.6%)	37	67/97 (69%)	71.1%	chr15:2,085,106–22,588,019. 1,735,914 bp	*OR4M2-OT1*(HGNC:56,199*), LINC02203* (HGNC:53,069*), OR4M2* (HGNC:15,373)*, OR4N4 (*NCBI: 283,694)*, OR4N3P* (HGNC:15,375)*, IGHV1OR15-1*(HGNC:5563)*, LOC102724760* (NCBI: 102,724,760)*, IGHV1OR15-3* (HGNC:5565)*, LOC642131* (NCBI: 642,131)*, MIR1268A* (HGNC:35,336)*, REREP3* (HGNC:38,797)
	3	24		23		30				
	4			2						

In region 2p11.2 for MXM healthy tissues, there was a gain of 3 and 4 copies, CN = 3 in 58% and CN = 4 in 41%, respectively, and only one case (0.67%) was CN = 2 (Fig. 2a); for MXM_A, 21% of cases presented with CN = 3 and 79% with CN = 4, with an overall copy gain across all groups of 99.6% (Fig. 2b, Table 2).

For chromosome 8p11.2, almost half of the cases, 42% of MXM and 51% of MXM_A, have CN = 2, but 49% MXM and 41% MXM_A have losses with CN = 1, and the rest (9% and 8%, respectively) have null CN = 0. The frequency of CN ≠ 2 across all the groups is 54.5% (Fig. 2, Table 2).

The CNV located in 14q32.33 is present as CN = 3 in 55% of MXM healthy samples and 62% in MXM_A samples and as CN = 4 in 45% of MXM and 38% of MXM_A subjects; there is not a single individual who shows CN = 2, 100% of individuals presented with gain in this region (Table 2, Fig. 2a and b).

Finally, in 15q11.2, approximately 70% of the samples had either gains or losses: 40% and 38% of the MXM healthy tissues and MXM_A samples, respectively, showed loss CN = 1, while 31.5% of MXM and 31% of MXM_A had gain with CN = 3, giving a frequency of CN ≠ 2 across all the groups of 71.1%.

The common CNV in MXM healthy subjects and tissues observed utilizing HapMap as a population of reference is shown in Fig. 3a.

### Validation of CN by qPCR

We performed qPCR to corroborate the CN obtained with GW SNP Array 6.0 and analyzed the gDNA for a representative sample of the individuals studied, including nine MXM individuals and seven MXM_A individuals. With this method, 15 out of 16 samples (93.75%) in *locus* 2p11.2 showed CN from 4 to 17 copies (ChAS only detects up to CN = 4). For chromosome 8, locus 8p11.22 A total of 14/16 (87.5%) samples showed < 2 copies. For 14q32.33, we corroborated the gain of the CNV in 13/16 (81.25%) with 3 to 5 copies; for chromosome 15, we found both losses and gains by GW, and we found 4 patients with loss and 6 with copy gains (25% and 37.5%, respectively) (Table 3, Fig. 3).

**Table 3:** qPCR validation of CNV found with GWAS SNP Array 6.0 in Mexican-Mestizo

ID	2p11.2 chr2: 89,143,755–92,057,597		8p11.22 chr8: 39,230,170–39,386,952		14q32.33 chr14: 106,078,230–107,100,266		15q11.2 chr15: 21,914,540–22,681,064	
Healthy	CN Array	CN qPCR	CN Array	CN qPCR	CN Array	CN qPCR	CN Array	CN qPCR
CtrNV03	3	2	1	1	3	2	2	2
CtrNV04	4	7	2	1	4	3	4	1
CtrNV05	3	4	2	1	3	2	2	2
CtrNV07	4	5	1	1	3	3	2	4
CtrNV08	4	6	1	1	4	3	1	2
CtrNV10	4	6	2	1	4	2	1	5
CtrNV11	4	4	1	1	3	3	3	4
CtrNV13	3	17	1	1	3	4	1	1
CtrNV16	4	5	2	1	4	3	1	1
Aneuploid								
NV20T13	4	7	1	1	4	2	3	4
NV31X0	4	4	1	1	3	3	2	ND
NV32T21	3	10	2	ND	3	3	1	ND
NV53T18	3	11	2	1	3	5	3	2
NV56T13	4	4	2	ND	3	ND	3	ND
NV60X0	4	10	2	2	3	5	1	1
NV62T18	4	7	1	1	4	ND	2	ND
NV115X0	4	ND	2	2	3	4	3	3
AE19T18	3	ND	2	ND	4	ND	1	1

*ND* No DNA available

### CRMA v2 analysis

The CNV found with the Chromosome Analysis Suite (ChAS) was validated with an additional CNV calling method CRMA v2.

We declare the raw data set conformed by 50 MXM CEL files (Genome-Wide Human SNP Array 6.0).

**Fig. 4:**
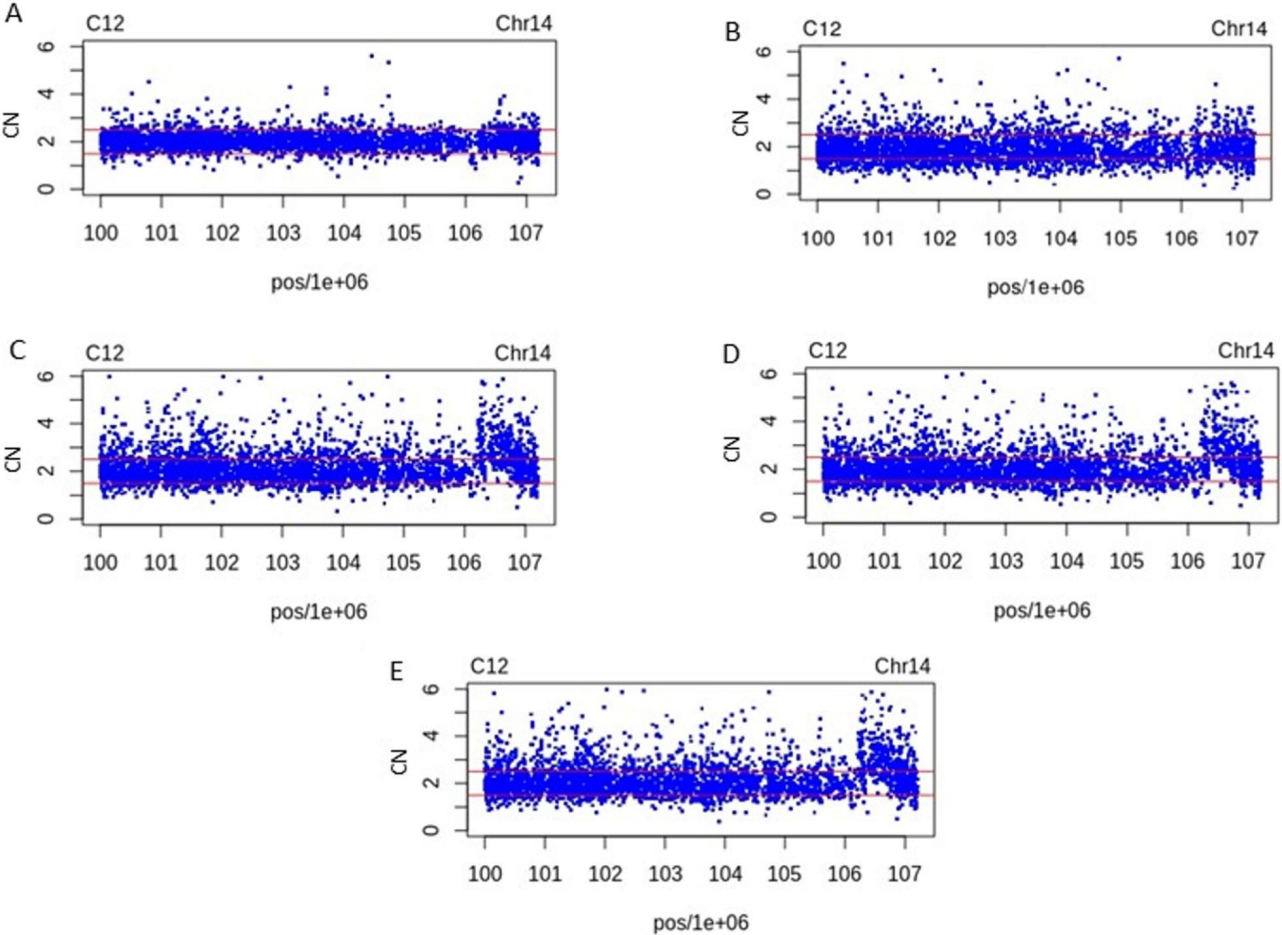
Copy number (CN) estimates in 14q32.33 for sample C12. **a** CN in the C12 vs. Mexican Mestizo Healthy (MXM) reference. **b** CN in the C12 vs. Iberian population in Spain (IBS) reference. **c** CN in the C12 vs. Caucasian (CEU) reference. **d** CN in C12 vs. Yoruba in Ibadan (YRI) reference. **e** CN in C12 vs. Hapmap reference (CEU + YRI). Note that the CNV in region chr14: 106,078,230–107,100,266 is only noticed when compared with HapMap CEU and YRI samples

This method could compare the copy number estimates in 14q32.33 for each sample. Figure 4 shows the CNV calling for chromosome 14 in subject C12 in the A) C12 *vs* MXM reference. B) C12 *vs* Spanish population (IBS) reference. C) C12 *vs* CEU reference. D) CN in the C12 vs. YRI reference. E) CN in C12 vs. two populations included in the HapMap reference (CEU + YRI). It is important to note that the difference in CN in our sample depends on the reference used.

It is clear that in A and B, the 14q32.33 region does not show changes with respect to the Mexican and Spanish reference population, emphasizing our Mestizo race formed by Spanish and Native American groups. In contrast, comparisons with CEU, YRI and CEU + YRI make evident a gain of probes located in the chr14:106,078,230–107,100,266 region, showing our racial differences with these populations.

## Discussion

In the tested samples, we found four high-frequency CNVs in the following *loci*: 2p11.2, 8p11.22, 14q32.33, and 15q11.2. No genes were found at 2p11.2 or 15q11.2, while the CNV at 8p11.22 included the genes *ADAM5* (HGNC: 212) and *ADAM3A* (HGNC: 209). The 14q32.33 *locus* comprises the genes *KIAA0125* (HGNC: 9834), *ADAM6* (HGNC: 8755), *LINC00226* (HGNC: 338,004), *IGHD* (HGNC:5480), *IGHG3* (HGNC:5527), and *IGHG1* (HGNC:5525); none of these genes has been associated with Mendelian diseases (OMIM) related to genetic dosage (https://search.clinicalgenome.org/kb/gene-dosage/region/ISCA-37477), and there are no regulatory sequences that could modify gene expression in any of the regions of the four CNV; neither of our CNV reported overlap with LCSH regions.

Additionally, by searching these specific regions in databases such as the sSMC (small supernumerary marker chromosomes) database http://cs-tl.de/DB/CA/sSMC/0-Start.html and the chromosomal heteromorphisms database http://cs-tl.de/DB/CA/HCM/0-Start.html, we found no evidence that a number other than CN = 2 in the 2p11.2, 8p11.22 and 15q11.2 regions or 14q32.33 duplications is associated with pathology, indicating that numerical variants in these regions have no impact on the phenotype, probably due to the absence of dosage-sensitive genes [25].

The high frequency of some variants could be due to the existence of inbreeding or consanguinity in the population. However, according to our results on the percentage of homozygosity of the LCSH regions and the degree of inbreeding (F value) [26], there is no indication of inbreeding or consanguinity in the studied groups.

CNVs are genomic variants that confer high variability among individuals and have been recognized for several years [1–3]. The first CNV studies were performed in the HapMap collection, which comprises 270 subjects divided into four populations: 90 Yoruba from Ibadan, Nigeria (YRI), 90 European descent from Utah, USA (CEU), 45 Japanese from Tokyo, Japan (JPT) and 45 Han Chinese from Beijing, China (CHB) [4]. In that study, the gDNA was analyzed with 500 K Affymetrix SNP arrays, and the CNV was determined using the software provided by Affymetrix. No individual presented CNVs at 2p11.2, 8p11.22, 14q32.33, or 15q11.2. In 2010, a study of large CNVs (1 kb) performed in 450 samples of HapMap (180 CEU, 180 YRI, 45 JPT and 45 CHB) reported CNV at 14q32.33 with a frequency less than 1% and in 2p11.2, 8p11.22 and 15q11.2 with a frequency near 5% [13].

Later in 2015, the phase 3 1000 genome study included 2504 healthy individuals, only 64 of them (2.5% of the total sample analyzed) were individuals of Mexican descent, and they all were living in Los Angeles, California (MXL) [18] This study in which Hispanic genomes were underrepresented showed low frequencies for CNVs at 2p11.2, 8p11.22, 14q32.33 and 15q11.2, coincident with the data previously reported by Conrad et al.[13].

Now we know that the CNV rate of occurrence has wide differences according to the ethnic group that is studied, as it occurs with other polymorphic traits such as SNPs or microsatellites [27]. Furthermore, frequencies may vary depending on the microarray platform used and the CNV calling algorithms [20, 28].

The results presented here are based on the Affymetrix microarray platform and were analyzed using Affymetrix software, with Affymetrix annotations NetAffx 33.1, which is the default software provided with this commercial platform. This workflow is the one being used to find CNV for clinical and research studies in Mexico and in several other countries, however, in this software there is little representation of the Latin American population.

In fact, there are studies reporting association of some of the polymorphisms studied, to pathological conditions [29–31]; one of these studies was conducted in Brazil and described the association of CNV gain of the 14q32.33 region with dental tumors [32], the reported copy gain in this 14q32.33 *locus* is as compared to the default reference genome without taking into account possible ethnic differences of the studied population, an ethnic matched reference genome would not have shown gain in this region. Indeed in 2020, Godoy et al. identified a CNV obtained from three different microarray platforms from a Brazilian population to conform the Brazilian CNV database. They found that a 14q32.33 gain was present in 97.8% of the samples studied [33], similar to that found in our study in 100% of the samples. Interestingly, the origins of the Brazilian population and ours have in common the mixture of the Iberian population that conquered us centuries ago.

The foregoing was addressed in this work by CNV calling (CRMA), where a Spanish population was used as reference, with this better-suited ethnical matched reference the gain of the CNV at 14q32.33 was reduced, and even did not appear as copy number gain (Fig. 4a and b).

The findings presented in this study contribute to the description of the frequency of common CNV in the Mexican-Mestizo population. Because microarrays are widely used tools in diagnostic and research contexts, this work is a clear example of why CNVs analysis must be carried out using an ethnically appropriate reference population. The significance of this is being recognized more wildly, as a consequence of this there is a conscient effort to include more diverse ethnic groups in the wildly used reference databases like HapMap [19]. This inclusive policy is fundamental to have a more accurate representation of the human genome.

## Conclusions

We found four CNVs, 2p11.2, 8p11.22, 14q32.33, and 15q11.2, with a high frequency in the Mexican-Mestizo population when contrasted with the HapMap population of reference (ChAS), while when using an ethnically related population as a reference, the differences were reduced or disappeared, highlighting the importance of analyzing the CNVs of the studied individuals with a reference group that (as far as possible) shares the same ethnicity. These findings contribute to a better description of the CNVs characteristics of the Mexican-Mestizo population and enhance the knowledge of genome variation in different ethnic groups.

## Methods

### Population, samples, and DNA extraction

We included blood samples from subjects with Mexican-Mestizo origin who consented to participating in this study. Fifty were healthy, unrelated volounteers from the general population. All subjects were over 18 years of age, had no history of genetic diseases and at the time of sample donation were not suffering from any infectious disease nor taking any medications (MXM, 30 females and 20 males). All participants had a normal karyotype 46,XX in females and 46,XY in males (20 corroborated by G banding in 25 metaphases, and 30 by chromosome microarray). We also included 97 aneuploid patients (MXM_A, 41 with trisomy 21, 28 with monosomy X, 11 with trisomy 13 and 17 with trisomy 18), with karyotype showing regular trisomy as well as X-monosomy without mosaicism, which was corroborated by interphase FISH (1000 cells analyzed).

Genomic DNA was extracted from the blood samples obtained from the participants with the saline precipitation method (Gentra Puregene Kit, QIAGEN, Venlo, Limburg, NL).

### Genome-wide Human SNP arrays

We analyzed the gDNA with the Affymetrix GeneChip® Genome-Wide Human SNP Array 6.0 (Santa Clara, CA, USA). This array contains 906,600 SNP probes and 946,000 nonpolymorphic oligonucleotides; the median intermarker distance over all 1.8 million SNP and copy number markers combined is less than 700 bases.

The procedures for DNA digestion, ligation, PCR amplification, fragmentation, labeling, denaturing and hybridization into the array were performed in 147 DNA samples (two DNA samples were not included because they did not pass quality controls) according to the protocols provided by the supplier. Arrays were stained and washed in the Affymetrix GeneChip Fluidic Station 450 and scanned using an Affymetrix GeneChip Scanner 3000 7G (Affymetrix, Santa Clara, CA, USA). We analyzed the files obtained with the appropriate bioinformatics tools.

### Long contiguous stretches of homozygosity

We visualized the long contiguous stretches of homozygosity (LCSH) in Chromosome Analysis Suite (ChAS) software version 4.1, provided by Affymetrix (Affymetrix, Santa Clara, CA, USA). For the analysis, we used the NetAffx 33 hg19 annotation files (http://www.affymetrix.com). For LCSH > 3 Mb, the analysis configuration was set at LOH with marker count = 50 and size = 3000 kb, and for LCSH > 5 Mb, it was set at marker count = 50 and size = 5000 kb.

### Estimation of the coefficient of inbreeding (F)

Individual inbreeding coefficients (F) were estimated using LCSH > 3 Mb data; F was the total length of autosomal LCSH in kb divided by the total autosomal size covered by the Genome-Wide Human SNP Array 6.0 (2,881,033,286 kb for hg19). We report the average of the F value for each group. An F value of 0.25 could reflect a first-degree parental relationship, 0.125 a second-degree relationship, 0.0625 a third-degree relationship and 0.03125 a fourth-degree relationship [26].

### Additional files

To compare our studied population, we used the following CEL files obtained from Affymetrix GeneChip® Genome-Wide Human SNP Array 6.0 (Santa Clara, CA, USA):

(a) Ninety-nine files from healthy tissues obtained from women with breast cancer (MXM_WBC) [34] from the GEO open database (GSE87048).

Together with the MXM, they will be the Healthy tissues.

(b) 30 Spanish (IBS) files downloaded from GEO data set GSE67047 [35]

(c) 30 Caucasian (CEU) and 10 Yoruba (YRI), part of the population studied in the HapMap project, CEL files kindly provided by Affymetrix/Thermo Fisher.

### Ancestry

The MAP and PED files obtained by Genotyping Console software (Affymetrix) were used for the ancestry analysis; we eliminated two files that did not pass quality control.

The global ancestry of 49/50 healthy Mexican-Mestizo files (MXM) and 96/97 individuals with aneuploidy (MXM_A) was deduced through a supervised maximum likelihood ADMIXTURE approach from K = 2 to K = 3 ancestral components and compared with the global ancestry of the group MXL (Mexican ancestry from Los Angeles California US) (n = 64). For this analysis, genotypic frequencies of Northern European (CEU) (n = 99), Yoruba (YRI) (n = 108), and Native American (NAT) (n = 43), reported in the 1000 genomes project (1KGP), were considered as reference parental populations for demographic and historical reasons.

#### Copy number variant (CNV) calling

CNVs were visualized with Affymetrix software (Affymetrix, Santa Clara, CA, USA). GeneChip Command Console (AGCC) software was used to generate the CEL files and ARR files from each microarray scanned. The CEL and ARR files were analyzed with Genotyping Console software (Affymetrix, Santa Clara, CA, USA) to obtain the CN data files (CNCHP) MAP and PED files for ancestry. CNCHP data were analyzed using Chromosome Analysis Suite (ChAS) software version 4.1 (Affymetrix, Santa Clara, CA, USA).

**Fig. 5:**
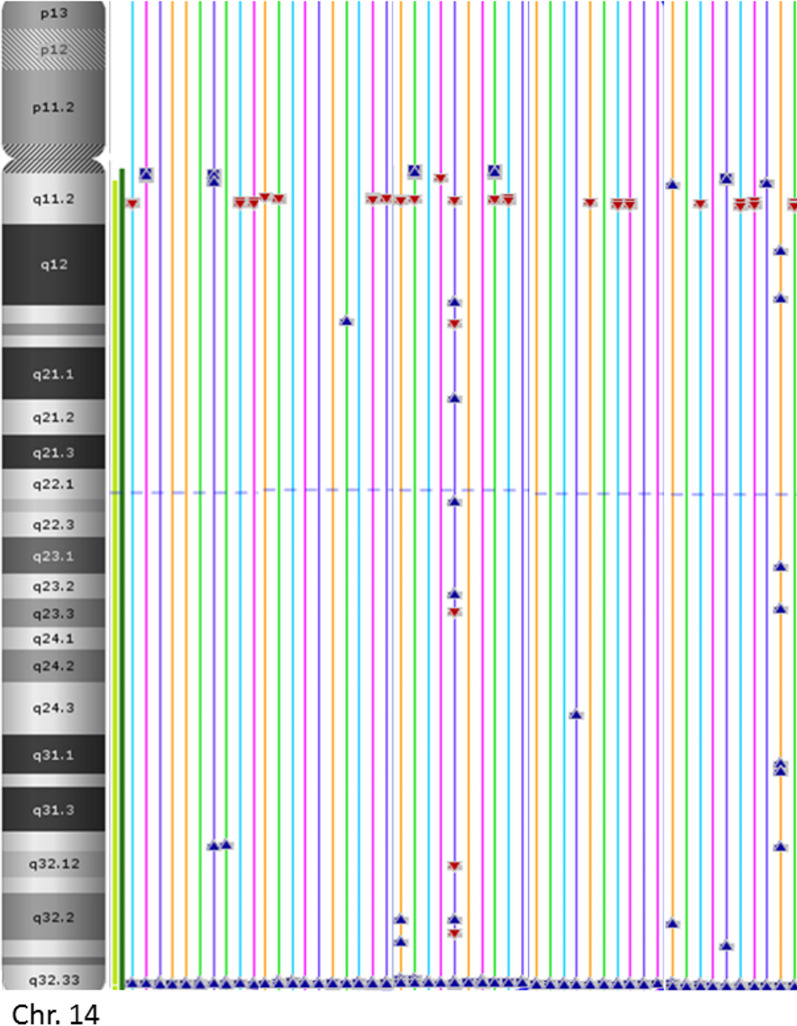
Chromosome 14 image with CNV highlighted, CN > 2 in blue arrowheads, and the regions with CN = 1 in red arrowheads, the CN regions with copy number (CN = 2) are only marked in lines. CNV in 14q32.33 is present in all samples of our group of study (n = 246). CN: Copy Number

The annotation file used in our analysis can be found on the Affymetrix website, listed as NetAffx 33.1 (hg19). The reporting threshold of the copy number was set at "High Resolution" settings, which gave us a total of gains or losses of 100 kb with a marker count ≥ 50. The copy number variants were compared among all samples. The CN analysis in ChAS software (http://www.affymetrix.com) has two ways of showing the results: one as a table that includes both coordinates and CN for each region; or a graphical interphase that displays the results of CN in an image of each of the 23 chromosomes, highlighting the regions with CN > 2 in blue arrowhead, regions with CN = 1 in red arrowhead, or CN regions with copy number (CN = 2), which are only marked in lines (Fig. 5).

To corroborate the changes in the CN obtained by GW SNP 6.0, we analyzed the absolute changes in copy number with qPCR in a representative sample. Oligonucleotides primers were designed at the four *loci* with CN ≠ 2 and for a distal *locus* on the same chromosome, where CN = 2 was used as a copy number control (Table 4). qPCR was performed with LightCycler ® Master (Roche Diagnostics) using hydrolysis TaqMan® probes (Universal Probes, Roche) with 100 ng of DNA, 45 amplification cycles and a single quantification. For those chromosomal regions in which it is possible to have a CN = 2 control DNA, the method proposed by Livak of 2^−DDCt^ was used [36]; for the *loci* where all subjects showed the change in the CN, the 2^−DCt^ method was used (Table 3) [36].

**Table 4:** Primers designed for qPCR of the *loci* with copy number variants (CNVs) and control regions

ID	3’–5' Sequence Forward	3’–5' Sequence Reverse
Chr2CNV	GGAAACACCTATTTGGACTGGT	GCCCGATAGGAAAGCGTATAG
Chr2Ctrl	TGATTTGGGTTGCACTTCTTT	TTGCAGCAAATAGGCGAATA
Chr8CNV	CAGCCGTTCCAAGGACAA	GAGACAGCATTGCGTAGCC
Chr8Ctrl	TGCAGTGAGCTCCCTAAGTCT	GCTCGGGAGTCTAACAGTCAA
Chr14CNV	AACACCCAGTGCAATGTGAC	TCCTCTATGACCGCACTTCTG
Chr14Ctrl	TTTTTGAAGGAGTTGGTTAAACATT	GTGTCCCTCAGCTAGGCAGT
Chr15CNV	TGAACAAGAGGGACAAGCAA	AGGGTATGTCCCCATCATCA
Chr15Ctrl	CAAAGTCTCCTAATCTTGGACAGC	GAGGGAAGACTAGGATGATACCTG

For an additional CNV calling method, we used copy number estimation using robust multichip analysis, and this method provides full-resolution raw total copy number estimates by preprocessing and probe summarization. CRMA v2 is available in Bioconductor [37] and implemented in R (http://www.aroma-project.org/vignettes/CRMAv2/).

Briefly, the CEL files were analyzed for quality after the program calibrated for crosstalk between allele probe pairs, followed by normalization for 25-mer nucleotide-position probe sequence effects. Next, we analyzed the performance of robust probe-summarization, normalized the PCR fragment-length effects on summary signals, and finally calculated the full-resolution total copy numbers. When a standard reference is used, it is often the average of a pool of samples CnChipEffectFile.

The reference control samples for CRMA v2 analysis were a) the same 50 healthy individuals of MXM; b) 30 IBS, 30 CEU and 10 YRI. All groups were analyzed with the same pipeline.

We made the following comparisons: (a) MXM vs. MXM reference; (b) MXM vs. IBS reference; (c) MXM vs. CEU reference; (d) MXM vs. YRI reference; and (e) MXM vs. HapMap reference (CEU + YRI).

### Databases

We reviewed publicly available databases to classify the CNVs found in our samples and the genes included:

Database of Genomic Variants (DGV, http://projects.tcag.ca/variation/).

DECIPHER (https://decipher.sanger.ac.uk).

ECARUCA (http://www.ecaruca.net/).

Genes2Cognition (http://www.g2conline.org/).

Ensembl (https://www.ensembl.org/index.html).

OMIM (https://www.omim.org/).

Gene ontology (http://geneontology.org/).

ClinVar (https://www.ncbi.nlm.nih.gov/clinvar/).

ClinGen (https://www.clinicalgenome.org/).

The sSMC database (http://cs-tl.de/DB/CA/sSMC/0-Start.html).

The heteromorphims database (http://cs-tl.de/DB/CA/HCM/0-Start.html).

https://franklin.genoox.com/clinical-db/home

## Data Availability

The data that support the findings of this study are not publicly available because one part of them is available from the Instituto Nacional de Ciencias Genómicas (México), but restrictions apply to the availability of these data, which were used under license for the current study. Data are, however, available from the authors upon reasonable request, with permission of Instituto Nacional de Ciencias Genómicas (México) and following the approved ethics committee guidelines.

## Abbreviations

CEU: Utah residents (CEPH) with Northern and Western European ancestry (1KGP population)
CHB: Han Chinese from Beijing, China
CN: Copy number
CNV: Copy number variant
DGV: The Database of Genomic Variants
Gb: Gigabases
GW SNP 6.0: Genome-Wide Human SNP Array 6.0, Affymetrix
hg19: Homo sapiens (human) genome assembly GRCh37
IBS: Iberian population in Spain
INP: Instituto Nacional de Pediatría
kb: Kilobases
LCSH: Long contiguous stretches of homozygosity
MAF: Minor allele frequency
MXL: Mexican Ancestry in Los Angeles CA, USA (1 KGP population)
MXM: Mexican Mestizo healthy controls
MXM_A: Mexican Mestizo with aneuploidy
MXM_WBC: Mexican-Woman with breast cancer (blood samples)
NAT: Native Americans (1KGP population)
OMIM: Online Mendelian Inheritance in Man
qPCR: Quantitative polymerase chain reaction
sSMC: Small Supernumerary Marker Chromosomes
SNP: Single nucleotide polymorphism
YRI: Yoruba in Ibadan, Nigeria (1KGP population)
